# When *HERG*-caused LQT2 encounters antisense oligonucleotide: is exon 6 skipping therapy plausible?

**DOI:** 10.3389/fphar.2025.1535259

**Published:** 2025-03-21

**Authors:** Zequn Zheng, Yongfei Song

**Affiliations:** ^1^ Ningbo Institute of Innovation for Combined Medicine and Engineering, Ningbo Medical Center Lihuili Hospital, Ningbo University, Ningbo, Zhejiang, China; ^2^ Department of Cardiology, First Affiliated Hospital of Shantou University Medical College, Shantou, Guangdong, China

**Keywords:** long QT syndrome type 2, hERG, nonsense-mediated mRNA decay, antisense oligonucleotide, exon skipping, therapy

## Abstract

**Figure:**
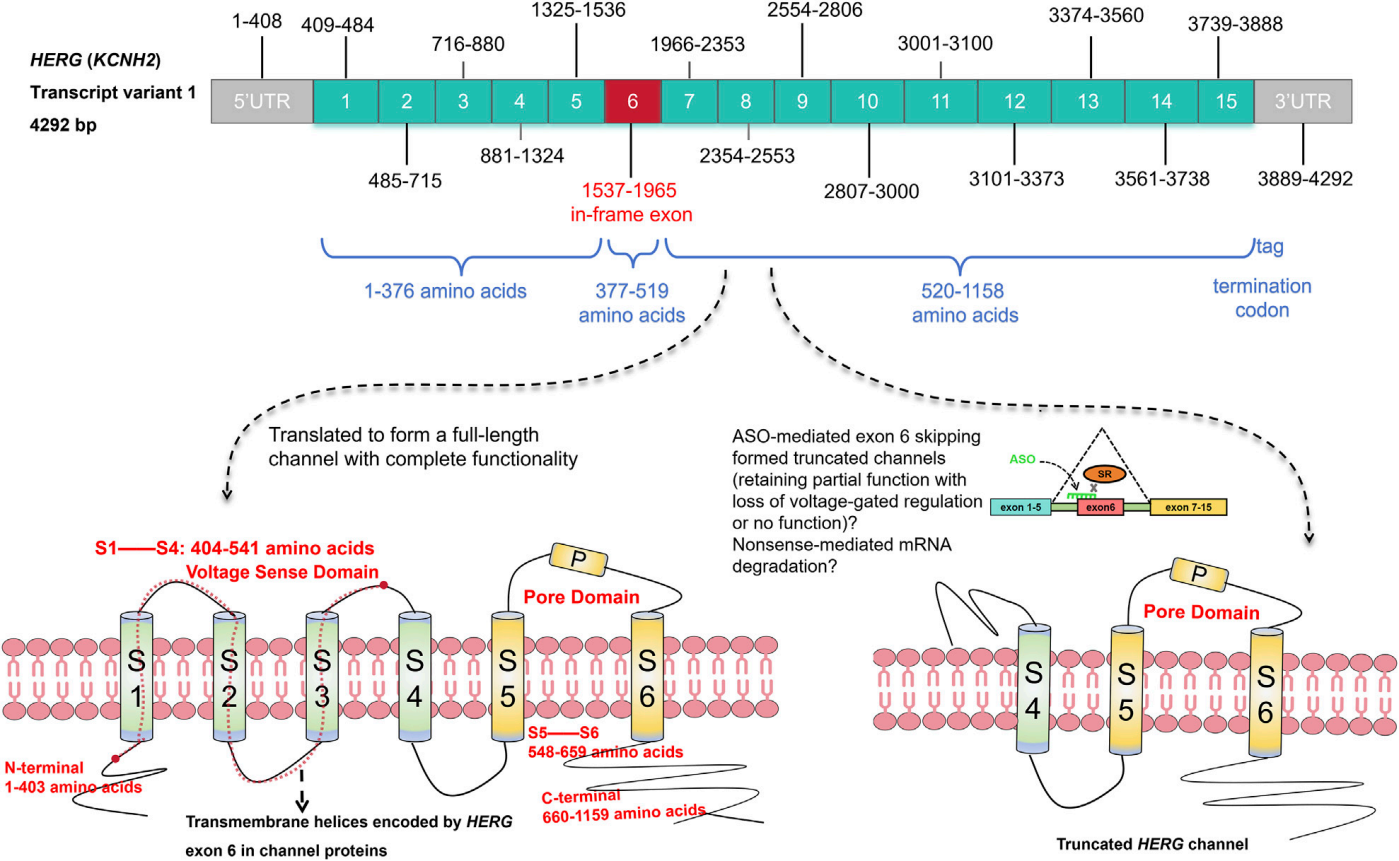
The unique in‐frame exon 6 of the HERG gene as a potential target for antisense oligonucleotide‐mediated exon skipping therapy.

The unique in‐frame exon 6 of the HERG gene as a potential target for antisense oligonucleotide‐mediated exon skipping therapy.

## Introduction

Long QT Syndrome (LQTS) represents a paradigmatic genetic arrhythmia with profound impacts on affected individuals, manifesting in syncope, seizures, and sudden cardiac death (Wang et al., 2023; Schwartz et al., 2020). Predominantly triggered by mutations in the human ether-a-go-go-related gene (*HERG*, also known as *KCNH2*), LQTS Type 2 (LQT2) represents a considerable fraction of LQTS cases (Schwartz et al., 2020; Wilde et al., 2022). The *HERG* gene encodes the Kv11.1 channel, crucial for the cardiac action potential’s repolarization phase, with mutations typically leading to potassium channel dysfunctions, thereby extending the cardiac action potential duration and precipitating lethal arrhythmias such as Torsades de Pointes (TdP) (Curran et al., 1995). Over 30% of *HERG* mutations result in premature termination codons (PTCs), inducing nonsense-mediated mRNA decay (NMD) and subsequently diminishing functional Kv11.1 channel expression (Gong et al., 2007). Recent advancements have illuminated antisense oligonucleotide (ASO)-mediated exon skipping as a strategic approach to bypass mutant exons, offering learnable and promising therapeutic avenues for LQT2 (Michaels et al., 2022; Kim et al., 2022; Oren et al., 2022). Building on comprehensive review we previously published (Zheng et al., 2023), this commentary explores the potential of targeting exon 6 within the *HERG* gene for therapeutic intervention, highlighting the scientific rationale, associated challenges, and future directions.

## The science behind exon skipping

ASOs are synthetic single-stranded oligonucleotides designed to hybridize with RNA transcripts, modulating pre-mRNA splicing. By masking splice sites, ASOs can effectively “skip” exons carrying pathogenic mutations, potentially restoring the open reading frame (ORF) and allowing for the production of partially functional proteins (Michaels et al., 2022). This innovative approach, previously applied in diseases like Duchenne Muscular Dystrophy, highlights its therapeutic possibilities (Young and Pyle, 2016). Recent foundational studies have applied exon skipping therapy to CFTR mutations causing cystic fibrosis, where ASO-mediated exon 23 skipping allowed the CFTR containing nonsense mutations to escape NMD, producing truncated amino acids with partial functionality (Michaels et al., 2022; Kim et al., 2022). For diseases caused by specific mutations, such as the exon 6 mutations in *HERG* associated with LQT2, the ideal ASO would specifically target transcripts from the mutant allele, allowing the wild-type allele to be expressed normally (Zheng et al., 2023).

## Targeting exon 6 in *HERG*

Exon 6 of the HERG gene is uniquely characterized as an “in-frame exon,” being the only one among the fifteen exons in the longest transcript variant (4,292 bp) that maintains these properties. It precisely spans amino acids 377 to 519 without the risk of frame-shifting mutations since it is encoded by a multiple of three nucleotides and does not share codons with adjoining exons. This specific exon is critical in encoding the N-terminal tail to the transmembrane fragment S3 of the channel, areas frequently associated with mutations leading to NMD, which significantly reduces HERG protein levels (Zequn and Jiangfang, 2021) (Graphic abstract). The potential skipping of exon 6 could theoretically preserve the ORF, allowing for the production of a Kv11.1 channel variant with sufficient functionality to possibly ameliorate the LQT2 phenotype. This strategy hinges on the ability of ASOs targeting exon 6 to enable HERG mRNA with nonsense mutations to bypass NMD, potentially producing a functional protein that could compensate for the channel deficiency. While there are concerns that evasion from NMD might lead to dominant negative effects due to the presence of defective HERG protein within cells, the generation of assuredly functional channels is crucial to address the haploinsufficiency observed in these cases (Anderson et al., 2006; Aizawa et al., 2023).

## Challenges in exon 6 skipping therapy

### Functional ambiguity

The exon-skipping therapy, while innovative, creates uncertainty about the function of the resulting protein. As mentioned above, exon 6 of the *HERG* gene contains essential structural domains critical for channel activation and inactivation gating kinetics. The integrity of these structural domains (such as the N-terminal tail, S1-S2 connector, and S3 helix) is critical for the proper functioning of the Kv11.1 channel (Zequn and Jiangfang, 2021). Excision of exon 6 while preserving the ORF may result in the deletion of critical regions, generating truncated channels that may possess compromised electrophysiological properties. Even without direct evidence showing the impact of entirely removing transmembrane helices S1–S3 on channel function, the potential to trigger enhanced channel activity via specific nonsense mutations within *HERG* exon 6 remains plausible (Zheng et al., 2023). However, it is important to consider that truncated channels may still retain minimal ion permeability or exhibit unconventional functionality under certain conditions. Although the absence of S1–S3 helices theoretically destabilizes the S4 helix in the membrane environment, the lipid bilayer or other molecular partners may partially stabilize its position. Existing studies also suggest that even structurally defective channels can occasionally form ion leak pathways, contributing to cellular ion flux (Zhorov and Tikhonov, 2024). Such observations imply that the absence of traditional voltage-gating properties does not necessarily result in a complete loss of function. Moreover, alternative mechanisms of functional compensation cannot be ruled out. Truncated channels might interact with endogenous proteins or leverage the membrane environment to stabilize partial functionality, such as stochastic ion permeation. While this remains speculative, there is no definitive evidence to exclude the possibility of residual activity. Interestingly, observations of channels with severed loop regions connecting core transmembrane segments, such as the S4-S5 linker, the S2-S3 intracellular loop, and the S3-S4 extracellular loop, have demonstrated that truncated proteins can, in some cases, retain functionality (de la Peña et al., 2018). Notably, it is unclear whether nonsense mutations in the exon 6 sequence induce NMD or produce truncated proteins. Therefore, it is essential to determine the susceptibility of these variants to NMD in the laboratory before adopting the exon-skipping strategy.

### Targeting and delivery hurdles

The specificity of ASOs to target only the mutant allele, sparing the wild-type gene, poses a significant challenge, especially in the heterozygous context common in LQT2. Off-target effects could lead to unforeseen consequences, including potential toxicity (Li et al., 2018). In LQT2, the pathogenicity often arises from a heterozygous mutation where one allele carries the mutation while the other remains unaffected. Ideally, an ASO therapy would modulate only the mutant allele’s expression to restore normal function without disrupting the wild-type allele’s beneficial effects. This precise targeting requires identifying unique markers or sequences associated with the mutation that are absent in the wild-type allele, a task that can be complex depending on the nature of the mutation.

To improve delivery, innovations in ASO chemistry, such as locked nucleic acids (LNAs) or peptide nucleic acids (PNAs), improve binding affinity and specificity while reducing the risk of degradation and immunogenicity (Egli and Manoharan, 2023; Baker et al., 2022). These modifications can help achieve the delicate balance between efficacy and safety. In addition, the high specificity of not needing a cocktail but a single ASO sequence to target the heart is a worthy endeavor.

### Long-term safety concerns

The prolonged impact of exon skipping remains largely unexplored. Continuous or repeated ASO application may cause cumulative toxicity, immune responses, or unforeseen genetic alterations (Egli and Manoharan, 2023). The potential for partially corrected channel function to induce substrates for arrhythmogenesis cannot be easily dismissed.

### Ethical and economic implications

Beyond scientific and technological challenges, exon 6 skipping therapy raises ethical concerns over patient selection, consent, and treatment access. Moreover, the high costs associated with ASO therapy may limit accessibility, highlighting fairness issues that necessitate concurrent resolution with scientific advancement.

## Comparison of exon skipping with other HERG rescue methods

The development of novel strategies to address *HERG* mutations responsible for LQT2 highlights the necessity of comparing ASO-mediated exon skipping with existing therapeutic approaches. Given the complexity and heterogeneity of *HERG* mutations, various methods such as small molecule therapies, allele-specific downregulation, allosteric modulator and translational readthrough agents have been explored (Sala et al., 2016; Yu et al., 2014; Zheng et al., 2022; Schwartz et al., 2019; Mehta et al., 2018; Lu et al., 2013). Each of these strategies presents unique strengths and limitations in addressing distinct mutation classes. ASO-mediated exon skipping is a targeted approach that holds promise for addressing Class 1 *HERG* mutations, particularly nonsense or frameshift mutations in exon 6. Unlike allele-specific downregulation strategies, which reduce dominant-negative effects but risk off-target impacts, inefficient delivery, and potential haploinsufficiency (Lu et al., 2013; Qu et al., 2023), ASOs offer high specificity with reduced systemic toxicity. Compared to wild-type protein enhancement, which compensates for loss of function but risks cytotoxicity and aggregation (Zhang et al., 2022), exon skipping provides a safer alternative. Small molecule therapies, like Lumacaftor, effectively address Class 2 trafficking defects but lack the precision needed for Class 1 mutations, while translational readthrough agents have shown limited success due to toxicity and variability (Yu et al., 2014; Schwartz et al., 2019; Mehta et al., 2018). Although ASO-mediated exon skipping excels in specificity, challenges remain in confirming truncated protein functionality, minimizing off-target effects, and ensuring long-term safety. A personalized, integrative approach combining exon skipping with other methods tailored to specific mutation classes offers the most promise for effectively treating LQT2.

## Future directions

Constructing a minigene plasmid lacking exon 6 but including introns and testing it in tool cells like HEK293 using techniques such as patch-clamping is a straightforward and rapid approach to discern whether the resulting channel produces current. This method also allows for the investigation of phenotypes associated with any point of interest involving nonsense mutations in this transgenic model. Importantly, the functional assessment of HERG channel proteins without exon 6 can be more accurately evaluated in an *in vitro* model that retains and generalizes the genetic context of the individual. In contrast to using tool cells for transgenic mimicry, the derivation of human induced pluripotent stem cells (hiPSCs) from LQT2 patients facilitates personalized disease models that mirror the genetic variability inherent in the condition. Cardiomyocytes derived from these patient-specific hiPSCs (hiPSC-CMs) offer valuable insights into the functional efficacy and safety of strategies like exon 6 skipping, highlighting the potential for tailored therapeutic interventions in the treatment of LQT2 (Song et al., 2022; Yu et al., 2023). It is particularly valuable for evaluating the efficacy of ASOs designed to skip exon 6 and their impact on cardiomyocyte function, as well as identifying any unintended cardiotoxic effects. Furthermore, developing targeted delivery systems to enhance cardiac cells’ ASO uptake while minimizing systemic exposure presents a promising avenue. Finally, thoughtfully designed clinical trials assessing both safety and efficacy are crucial, incorporating endpoints that reflect meaningful clinical benefits for patients.

## Conclusion

ASO-mediated exon 6 skipping in LQT2 marks a frontier in gene therapy, potentially offering targeted treatments for genetic arrhythmias caused by *HERG* mutations. However, this promising avenue is not without its significant challenges. Some of our colleagues in the field of *HERG* research would firmly argue that channels lacking the coding structural domain of exon 6 are inherently nonfunctional. We contend against such a definitive stance, as it aligns with Schrödinger’s cat principle: until experimentally confirmed, a critical yet cautiously optimistic perspective is essential—similar to the quantum mechanics thought experiment where a system exists in multiple states until observed, here too, the functionality of channels without exon 6 exists in a state of potential efficacy and inefficacy until definitive experimental evidence is available. We emphasize the need to balance optimism with rigor as we advance, ensuring that the pursuit of innovative therapies is grounded in ethical principles and a commitment to patient safety. This commentary also encourages researchers in the field of LQT2 to entertain the ideas presented here and push the boundaries of innovation further. We salute those contributing to research on LQT2 caused by *HERG* mutations and extend our gratitude to the reviewers of this manuscript.
